# Insights into Microbial Community and Its Enzymatic Profiles in Commercial Dry-Aged Beef

**DOI:** 10.3390/foods14030529

**Published:** 2025-02-06

**Authors:** Yinchu Liu, Xiaoguang Gao, Mingwu Zang, Baozhong Sun, Songshan Zhang, Peng Xie, Xiaochang Liu

**Affiliations:** 1 State Key Laboratory of Animal Nutrition and Feeding, Institute of Animal Sciences, Chinese Academy of Agricultural Sciences, Beijing 100193, China; liuyinchu88@163.com (Y.L.); zangmw@126.com (M.Z.); baozhongsun@163.com (B.S.); zhangsongshan_1997@163.com (S.Z.); seulbird@163.com (P.X.); 2 College of Food Science and Biology, Hebei University of Science and Technology, Shijiazhuang 050018, China; gaoxiaoguang23@hotmail.com

**Keywords:** dry aging, bacterial community, fungal community, *Pseudomonas*, *Candida sake*, *Candida zeylanoides*

## Abstract

Dry-aged beef has gained interest worldwide in recent years due to its improved sensory attributes. This enhancement is thought to be partially driven by microbial activities, particularly lipolysis and proteolysis. In this study, dry-aged beef manufactured by seven commercial producers in China was analyzed. The pH value and total volatile base nitrogen value of dry-aged beef were determined. High-throughput amplicon sequencing of full-length 16S rRNA genes and internal transcribed spacer (ITS) regions was used to analyze the microbial community. A total of 207 proteolytic and lipolytic isolates were identified by sequencing 16S rRNA genes for bacteria and sequencing the D1/D2 region of 28S rRNA genes and the ITS region for fungi. The results revealed that the crust harbored greater numbers of bacteria and fungi than the interior. The bacterial community was dominated by *Pseudomonas* species, which were core members in both the crust and interior, while *Brochothrix thermosphacta* was identified as a core bacterium exclusively in the crust. The fungal community primarily constituted *Candida sake*, *Kurtzmaniella* species, and members of the phylum Chytridiomycota. Proteolytic and lipolytic isolates were mainly identified as *Pseudomonas* sp., *B. thermosphacta*, *Carnobacterium maltaromaticum*, *Candida zeylanoides* (teleomorph: *Kurtzmaniella zeylanoides*), *C. sake*, and *Debaryomyces hansenii*. Two strains of *C. zeylanoides* and *C. sake* exhibiting high proteolytic and lipolytic activities effectively hydrolyzed beef fat, myofibrillar protein, and sarcoplasmic protein. This study characterized the main microorganisms and their enzymatic functions associated with dry-aged beef, highlighting the need to explore their contributions to the sensory attributes of dry-aged beef.

## 1. Introduction

Dry aging is a method for preserving unpackaged meat under chilled conditions with controlled temperature and relative humidity for several weeks. This process improves meat tenderness and develops distinctive flavors, such as nutty, buttery, and mushroom-like notes, driven by microbial activity, enzymatic activity, oxidation, and dehydration [1,2]. During dry aging, the direct exposure to the environment causes surface dehydration of the beef, forming a crust that may harbor large numbers of bacteria and fungi in some cases [3,4,5]. Many researchers suggest that the microbiota in dry-aged beef may contribute to changes in flavor profiles and other quality characteristics [6,7]. Thus, a comprehensive understanding of the microbiota in dry-aged beef is essential. To date, studies on the microbiota of dry-aged beef have been conducted in the United States [8], Belgium [4], and France [5], but similar investigations in China are rare.

Proteolysis and lipolysis are recognized as the most important microbial enzymatic activities influencing the quality of dry-aged beef. Fungal lipolytic and proteolytic activities have been shown to produce taste- and aroma-active compounds that enhance the sensory quality of beef. For instance, *Pilaira anomala* and *Debaryomyces hansenii*, isolated for their strong abilities to secrete protease and lipase, have been demonstrated to produce free amino acids and free fatty acids in dry-aged beef [9]. Similarly, Chen et al. [10] reported that *Debaryomyces hansenii* and *Pediococcus pentosaceus* degraded proteins to produce free amino acids, contributing to improved flavor in beef. Moreover, Przybylski et al. [11] found that *Mucor flavus* degraded myosin light chains and improved sensory attributes including the odor, softness, and juiciness of dry-aged beef. In contrast to these beneficial contributors, many proteolytic and lipolytic microorganisms are implicated in meat spoilage. Species in the genera *Serratia* and *Pseudomonas* are known to aggressively degrade meat proteins and function as specific spoilage organisms [12]. Thus, examining the proteolytic and lipolytic abilities of microbiota from dry-aged beef is essential for identifying microorganisms that either enhance or comprise the quality of dry-aged beef.

This study aimed to determine the microbial loads and composition present in dry-aged beef as well as their enzymatic capabilities. Microbial communities from commercial dry-aged beef samples collected from seven producers were analyzed. To achieve a high taxonomic resolution, high-throughput amplicon sequencing of the full-length 16S rRNA gene and ITS region was performed to determine bacterial and fungal communities, respectively. Proteolytic and lipolytic activities of microorganisms were assessed using the agar diffusion assay, and positive isolates were further identified.

## 2. Materials and Methods

### 2.1. Sample Collection

Dry-aged beef strip loins (boneless and with a fat cover) were sampled from seven commercial companies across China (Producer A, B, C, D, E, F, and G). All loins had been dry-aged for 42 days. Approximately two kilograms of each loin (without trimming) were sampled, placed in sterile bags, and transported to the laboratory using insulated boxes with ice packs. Samples collected from location C had been trimmed by the producer before our sampling. Three loins were collected from each producer. Environmental parameters were collected through direct inquiries with the producers (Table 1).

### 2.2. Determination of Physicochemical Parameters

The pH value and total volatile base nitrogen (TVB-N) value of trimmed beef were determined. The pH value was measured using a pH meter (Hanna, Woonsocket, RI, USA), attached with a penetration probe. The TVB-N value was determined using the method described by Zhang et al. [13]. TVB-N contents were expressed as mg/100 g of flesh.

### 2.3. Determination of Microbial Counts

Total aerobic bacterial counts and fungal counts in the crust and interior of dry-aged beef were determined. The crust (5 cm × 5 cm in area, approximately 2 mm in thick) was removed using sterile knives and forceps in an ultra-clean bench. For the inner part, muscle (approximately 5 mm in thick) was removed from the exposed area and used for microbial analysis. This study focused on the microbiota near the crust of dry-aged beef, where microbial diversity tends to be more abundant. For samples from producer C, 10 g of muscle was taken from the surface of the trimmed loin and was taken as the interior part. The crust or interior samples were homogenized with a 9-fold (*w*/*v*) sterile saline solution (0.9%, *w*/*w*) for 3 min in a stomacher (IUL, Barcelona, Spain). The mixture was 10-fold serially diluted and used for microbial analysis. Total aerobic bacteria were cultivated on plate count agar (PCA) (Solarbio, Beijing, China) and incubated at 20 ± 1 °C for 3 days. Total fungal counts were cultivated on potato dextrose agar (PDA) supplemented with 0.1% chloramphenicol (Solarbio, Beijing, China) and incubated at 20 ± 1 °C for 5 days. Microbial counts were expressed as log10 cfu/g.

### 2.4. Analysis of Microbial Community

#### 2.4.1. Microbial DNA Extraction and Sequencing

Total microbial DNA was extracted from the crust and interior of dry-aged beef using the E.Z.N.A.^®^ Soil DNA Kit (Omega Bio-tek, Norcross, GA, USA). The DNA quality was determined using 1% agarose gel electrophoresis, and the concentration was determined using NanoDrop 2000 (Thermo Fisher Scientific, Waltham, MA, USA). Bacterial 16S rRNA genes were amplified using the primers 27F (5′-AGRGTTYGATYMTGGCTCAG-3′) and 1492R (5′-RGYTACCTTGTTACGACTT-3′). Fungal ITS genes were amplified using the primers ITS1F (5′-CTTGGTCATTTAGAGGAAGTAA-3’) and ITS4R (5′-TCCTCCGCTTATTGATATGC-3’). PCR products were purified using AMPure^®^ PB beads (Pacific Biosciences, Menlo Park, CA, USA) and quantified with Qubit 4.0 (Thermo Fisher Scientific, Waltham, MA, USA). The purified products were pooled. The DNA library was constructed using the SMRTbell prep kit 3.0 (Pacific Biosciences, Menlo Park, CA, USA) following PacBio’s instructions. The purified SMRTbell libraries were sequenced using the PacBio Sequel IIe System (Pacific Biosciences, Menlo Park, CA, USA) by Majorbio Bio-Pharm Technology Co., Ltd. (Shanghai, China). High-fidelity (HiFi) reads were obtained from the subreads generated by circular consensus sequencing (SMRT Link v11.0).

#### 2.4.2. Bioinformatic Analysis

HiFi reads were identified using barcodes and filtered based on their length. For bacterial 16S rRNA genes, sequences with a length of <1000 bp or >1800 bp were removed. For fungal ITS genes, sequences with a length of <300 bp or >900 bp were discarded. The optimized HiFi reads were denoised to obtain amplicon sequence variants (ASVs), using the DADA2 algorithm on the QIIME2 platform. These ASVs were subsampled to match the lowest sequence count per sample, and any ASVs occurring below 0.2% incidence in at least two samples were removed. The taxonomic assignment of each ASV was performed using the Nucleotide Sequences Database for the bacterial biota and the Unite Database (version 8.0) for the mycobiota. The taxonomic assignment was manually cross-verified using the Basic Local Alignment Search Tool (BLAST). The sequencing data were deposited in the NCBI Sequence Read Archive and are available under the Bioproject Accession Number [PRJNA1028106].

### 2.5. Determination of Proteolytic and Lipolytic Activities

After microbial enumeration, colonies on PCA and PDA were selected by size, color, gloss, and transparency and were streaked to purify. The proteolytic and lipolytic activities of purified isolates were first evaluated by agar diffusion assays. Yeast strains that exhibited high proteolytic and lipolytic activities were cultured in separate systems containing sarcoplasmic protein, myofibrillar protein, and fat to evaluate their capacities to degrade beef protein and fat.

#### 2.5.1. Enzymatic Activities in Culture Media

Proteolysis was determined on PCA or PDA containing 1% (*w*/*v*) skimmed milk powder. The proteolytic activity was indicated by the presence of a transparent halo around the colony. The ability to hydrolyze triacylglycerols of short-chain fatty acids was tested on PCA or PDA containing 1.5% tributyrin emulsified with a 0.18% (*w*/*v*) gum Arabic solution. The presence of a transparent halo indicated lipolytic activity. The ability to hydrolyze triacylglycerols of long-chain fatty acids was determined on PCA or PDA containing 2.5% olive oil (substrate) and 1% rhodamine B (indicator). An orange fluorescent halo (under 350 nm) around the colony indicated lipolytic activity. All plates were incubated at 20 ± 1 °C for 5 days. Enzymatic activity was represented by the ratio of the diameter of the halo to the diameter of the colony.

#### 2.5.2. Enzymatic Activities in Systems Containing Beef Protein or Fat

Sarcoplasmic and myofibrillar proteins were extracted by the following process. Beef was homogenized with 10-fold volumes (*w*/*v*) of potassium phosphate solution (0.02 mol/L, pH 6.5) and centrifuged. The supernatant was collected as the sarcoplasmic protein. The pellet was washed three times with wash buffer (0.1 mol/L of NaCl, 0.02 mol/L of potassium phosphate, pH 6.5). Myofibrillar protein in the pellet was resuspended in 9-fold volumes (*w*/*v*) of the buffer (0.6 mol/L of NaCl, 0.1 mol/L of potassium phosphate, pH 6.5) and centrifuged to obtain the supernatant. The sarcoplasmic and myofibrillar proteins were filtered through 0.45 μm sterile membranes.

The protein-based model system was prepared by dissolving autoclaved glucose solution (final concentration of 1%, *w*/*v*) in the sarcoplasmic or myofibrillar protein solution. Overnight cultures (approximately 8.0 log10 cfu/mL) were inoculated into the model system. After incubation, the culture was mixed with a 3-fold trichloroacetic acid solution (TCA, 20%, *w*/*v*) to precipitate protein. The contents of peptides and free amino acids (FAAs) in the supernatant were determined by the BCA protein content kit (Solarbio, Beijing, China) and the amino acid content kit (Solarbio, Beijing, China), respectively. For the preparation of the beef fat-based model system, melt backfat (1.5%, *w*/*v*) was homogenized with PDA and autoclaved. Five hundred milliliters of the mixture was added to each well of the 24-well plate and left to solidify. Overnight cultures were spread on the surface of the model system. After incubation, all media were removed from each well and used for the determination of free fatty acids (FFAs) by a Non-esterified Free Fatty Acid colorimetric test kit (Elabscience Biotechnology, Wuhan, China). Contents of FFAs in the model system were expressed as μmol/g. All incubation was conducted at 20 °C for 5 days. Un-inoculated systems were also prepared to monitor autolytic proteolysis or lipolysis.

### 2.6. Identification of Proteolytic and Lipolytic Microorganisms

All proteolytic or lipolytic isolates were identified by gene sequence analysis. For bacteria isolates, the 16S rRNA genes were amplified using universal primers: 27F (5′-AGAGTTTGATCCTGGCTCAG-3′) and 1492R (5′-GGTTACCTTGTTACGACTT-3′). For fungi isolates, the internal transcribed spacer (ITS) region and D1/D2 regions of 28S rRNA genes were amplified using the primer pairs ITS1 (5′-TCCGTAGTGAACCTGCGG-3′) and ITS4 (5′-TCCTCCGCTTATTGATATGC-3′), and NL1 (5′-GCATATCAATAAGCGGAGGAAAAG-3′) and NL4 (5′-GGTCCGTGTTTCAAGACGG-3′), respectively. PCR products were sequenced by SinoGenoMax Co., Ltd. (Beijing, China). To identify the closest relatives, the 16S rRNA gene sequences were aligned with the EzTaxon database (https://www.ezbiocloud.net, accessed on 10 April 2023), and the ITS and D1/D2 gene sequences were aligned with the NCBI database (https://www.ncbi.nlm.nih.gov/BLAST, accessed on 10 April 2023). Sequences with greater than 97% identity were classified at the species level, while those identified as *Pseudomonas* were assigned at the genus level. 

### 2.7. Statistical Analysis

The data were expressed as the mean ± standard deviation. Significant differences were assessed using Duncan’s multiple comparisons in a one-way analysis of variance utilizing SPSS version 26 (SPSS, IBM, Chicago, IL, USA). *p*-values below 0.05 were considered statistically significant. Permutational Multivariate Analysis of Variance (PERMANOVA) was conducted using amplicon sequence variant (ASV) count data on the online tool of Majorbio Cloud Platform (https://cloud.majorbio.com/page/tools/, accessed on 10 April 2023). Violin plots were generated using GraphPad Prism 9.4.1 (GraphPad Software INC., La Jolla, CA, USA). The Upset map plots were produced using an online tool (https://www.chiplot.online/, accessed on 10 April 2023). The interaction network of microbiota and environmental parameters was calculated using the Mantel test and Pearson correlation coefficients using R 4.4.1 and package linkET 0.0.7.4.

## 3. Results and Discussion

### 3.1. Physicochemical Parameters

As shown in Table 2, the pH values of dry-aged beef ranged from 5.58 to 5.78. The TVB-N values of dry-aged beef varied with producers, ranging from 10.12 to 19.41 mg/100 g. TVB-N is produced by microbial and enzymatic activities and serves as an indicator of meat freshness. The wide range of TVB-N might reflect differences in the degree of beef freshness. Studies by Zhang et al. [13] and Kang et al. [14] reported that TVB-N values in dry-aged beef increased with prolonged aging time. Freshness thresholds for TVB-N vary across studies, with common limits set at 15 mg/100 g or 20 mg/100 g [15]. In this study, only one sample from producer E slightly exceeded the threshold, with a TVB-N value of 20.4 mg/100 g.

### 3.2. Microbial Counts

As shown in Table 3, total aerobic bacteria counts in the crust of 42-day dry-aged beef ranged from 6.2 to 8.8 log_10_ cfu/g, while fungi counts ranged from 5.3 to 7.7 log_10_ cfu/g. Previous studies have reported bacterial levels of 4–7 log_10_ cfu/g and fungal levels of 5–8 log_10_ cfu/g in the crust of beef dry-aged for 35–49 days [3,4,5,16]. Microbial levels in the crust are influenced by several factors, including the initial microbial quality of the raw meat, hygiene practices, environmental conditions (e.g., relative humidity, temperature, and air viscosity), and aging duration. Although low temperatures and reduced water activity in the crust inhibit bacterial growth during dry aging, the European Food Safety Authority Panel on Biological Hazards [17] advises minimizing the aging duration to limit spoilage bacterial proliferation, provided that desired organoleptic characteristics are maintained.

The interior of dry-aged beef from six producers (excluding producer C) carried lower bacterial and fungal loads compared to the crust, with bacterial counts ranging from <2.0 to 6.3 log_10_ cfu/g and fungal counts from <2.0 to 5.5 log_10_ cfu/g. For samples from producer C, bacterial and fungal counts in the interior were 7.1 log_10_ cfu/g and 4.7 log_10_ cfu/g, respectively. As the samples from producer C were trimmed under its routine practices, the data of producer C may reflect real production processes. Microorganisms detected in the interior of dry-aged beef may originate through three primary pathways: (i) migration from the outer crust during aging, (ii) proliferation of microorganisms initially present in the meat prior to aging, and (iii) contamination during trimming or sampling.

### 3.3. Microbial Community

The crust of dry-aged beef samples from seven producers exhibited high levels of bacteria and fungi. Although the crust of dry-aged beef is typically trimmed before sale, the microbial community within it can affect the interior quality by migrating inward or secreting enzymes that penetrate the meat. While bacterial and fungal counts in the interior were relatively low, the microbial community in the interior remains important due to its potential for proliferation during extended aging and storage. Thus, understanding the microbial ecology of both the crust and interior is essential for characterizing quality formation during dry aging and for maintaining quality during storage.

#### 3.3.1. Bacterial Community

**Figure 1:**
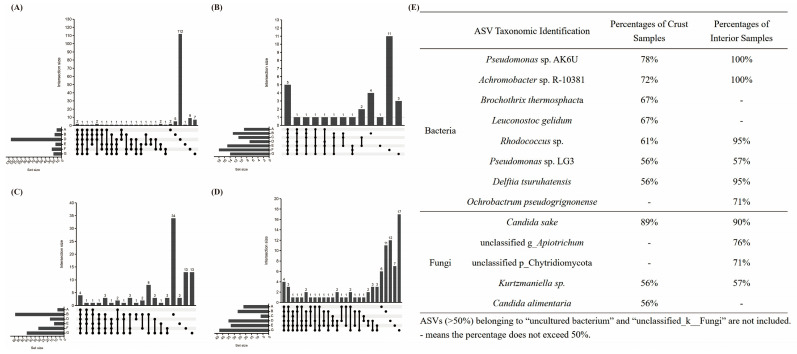
The Upset map of the unique and coincident ASVs in dry-aged beef from seven producers: (**A**) Bacteria ASVs in the crust, (**B**) bacteria ASVs in the interior, (**C**) fungi ASVs in the crust, (**D**) fungi ASVs in the interior, and (**E**) fungi and bacteria ASVs identified in more than 50% of crust or interior samples.

A total of 722,475 high-quality reads were obtained from 21 crust samples and 18 interior samples, with an average read length of 1500 bp. These reads were clustered into 265 ASVs. Good’s coverage values across all samples exceeded 99.9%, suggesting that the sequencing depth was sufficient to capture the bacterial diversity present. According to the Alpha diversity analysis, the Chao1 index ranged from 22 to 116 in the crust and from 12 to 25 in the interior, while the Shannon index varied from 1.559 to 2.921 in the crust and from 1.016 to 1.890 in the interior. Beta diversity analysis using PERMANOVA at the ASV level revealed significant differences in bacterial communities among producers (*p* = 0.001, R^2^ = 0.50) and between the crust and interior (*p* = 0.011, R^2^ = 0.04). The common and unique number of bacteria ASVs in the crust of dry-aged beef are depicted in the Upset map (Figure 1A,B). Meanwhile, core bacteria ASVs, defined as those detected in at least 50% of crust or interior samples and contained a minimum of 10 reads per sample, are summarized in Figure 1E.

**Figure 2:**
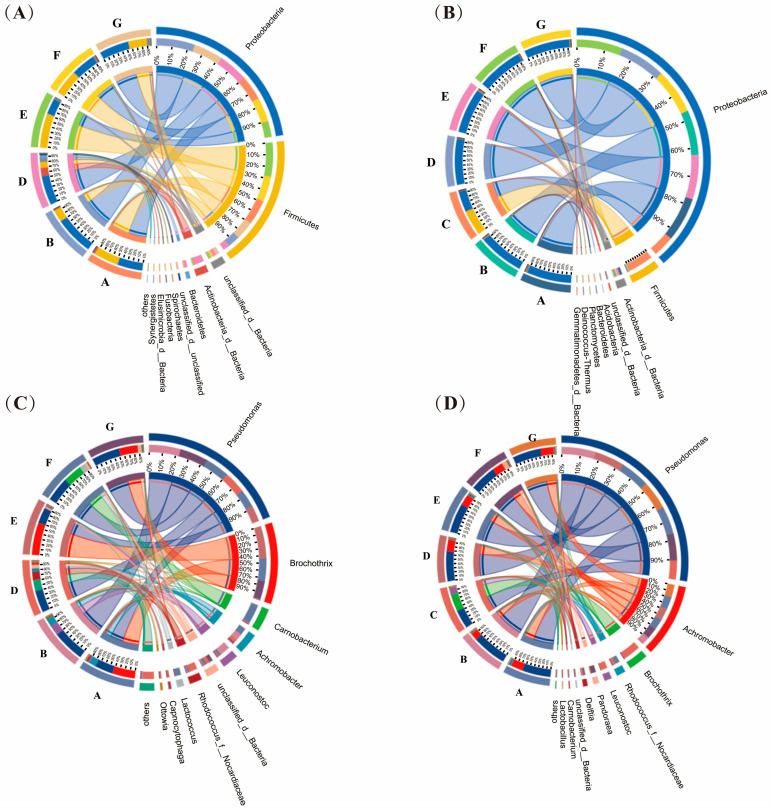
Circos plots of bacterial community in dry-aged beef. (**A**) Bacterial phyla in the crust, (**B**) bacterial phyla in the interior, (**C**) bacterial genera in the crust, (**D**) bacterial genera in the interior. The letters (A, B, C, D, E, F, and G) in each circos plot represent different producers. Each plot displays the top 10 taxa.

The bacterial composition of dry-aged beef is shown in Figure 2. *Pseudomonas* was highly abundant in both the crust and interior of samples from all producers. Two ASVs assigned to distinct *Pseudomonas* species were core ASVs, appearing in 78% and 100% of crust samples and 56% and 57% of interior samples (Figure 1E). Although high-throughput sequencing of the complete 16S rRNA gene provides a high taxonomic resolution for bacterial communities, the species-level classification of *Pseudomonas* remains challenging due to the highly conserved nature of its 16S rRNA genes [18]. Consequently, *Pseudomonas* was not classified at the species level in this study. Previous studies have showed that many *Pseudomonas* species can penetrate and migrate into meat tissues due to their high motility and proteolytic activity, which enables them to degrade connective tissue between muscle fibers [19,20]. These abilities may explain the high prevalence of *Pseudomonas* in the interior of dry-aged beef. The prevalence and dominance of *Pseudomonas* in dry-aged beef have been documented by Capouya et al. [8] and Coton et al. [5]. Several *Pseudomonas* species, including *Pseudomonas fragi*, *Pseudomonas fluorescens*, and *Pseudomonas putida*, are frequently associated with meat spoilage, leading to texture softening, off-flavor, sliminess, and discoloration [12]. Studies have demonstrated that antibacterial chitosan films [13] and probiotic *Lactobacillus* strains [21] could effectively control *Pseudomonas* in dry-aged beef and maintain beef quality.

A core ASV, identified as *Achromobacter* sp. R-10381 appeared in 72% of crust samples and all interior samples (Figure 1E). This ASV constituted high proportions, ranging from 7% to 14% in the crust of samples from producers A, B, and D, and from 17% to 30% in the interior of samples from producers A, B, D, E, F, and G. *Achromobacter* is frequently detected in chilled fresh meat [22], but its specific role and association with meat is poorly understood.

*Brochothrix thermosphacta* was highly abundant (>35%) in the crust of samples from producers A (43.4%), E (62.2%), and G (35.5%) but was scarce (<0.5%) in the interior of their counterparts. Nearly all ASVs (101,277/101,281) assigned to *Brochothrix* were identified as *B. thermosphacta* at the species level. The absence of *B. thermosphacta* in the interior may be attributed to its poor growth in the oxygen-deficient environment of the interior, or its inability to penetrate deep meat tissues. Although *B. thermosphacta* is a facultative anaerobic bacterium, it grows well only in the presence of sufficient oxygen [23]. Interestingly, a large proportion of *Brochothrix* was detected in the interior of samples from producer C. The large number of *Brochothrix* may be attributed to contamination, as samples from producer C were trimmed by the producer, and hygiene was not guaranteed during the process. EFSA BIOHAZ Panel [17] emphasized that cutting and trimming must follow strict hygienic procedures to prevent microbial contamination.

*Leuconostoc gelidum*, *Rhodococcus* sp., *Delftia tsuruhatensis*, and *Ochrobactru pseudogrignonense* were also detected as core species. These species were generally present at very low relative abundances, except for *L. gelidum*, which accounted for 20.3% in the crust of samples from producer B. *L. gelidum* is recognized as a spoilage bacterium in meat and is associated with the production of buttery and sour off-odors [24]. Additionally, certain bacterial species exhibited high relative abundances exclusively in specific samples, such as *Carnobacterium maltaromaticum* and *Lactococcus piscium* in the crust of samples from producer F.

In general, *Pseudomonas* and *B. thermosphacta*, both common spoilage bacteria in aerobically stored meat, were also frequently detected in dry-aged beef. However, their spatial distribution in dry-aged beef differed from that observed in aerobically stored meat. *Pseudomonas* was dominant in both crust and interior, while *Brochothrix* was frequently and abundantly identified only in the crust. This distribution may be attributed to the crust forming an air-restricted environment, which limits the growth of certain bacteria in the interior of the meat. The activities of those bacteria in the dry-aging condition need be considered.

#### 3.3.2. Fungal Community

From a total of 39 samples, 342,147 high-quality reads were obtained and were clustered into 559 unique ASVs. The average read length was 592 bp. Good’s coverage values were above 99.9% for each sample, confirming that the sequencing depth was sufficient to capture the existing fungal diversity. The Chao1 index of the fungal community ranged from 13 to 56 in the crust and from 14 to 68 in the interior, while the Shannon index varied from 0.762 to 2.646 in the crust and from 1.197 to 3.336 in the interior. The PERMANOVA of fungal community at the ASV level showed significant differences among producers (*p* = 0.001, R^2^ = 0.29) and between the crust and interior (*p* = 0.011, R^2^ = 0.23). Despite these differences, several fungal ASVs were shared across producers and sections (Figure 1C,D). Core fungal ASVs included *Candida sake*, *Candida alimentaria*, *Kurtzmaniella* sp., and two unclassified ASVs, one belonging to the genus *Apiotrichum* and the other to the phylum Chytridiomycota (Figure 1E). The results differed from those reported in the United States and France. *Debaryomyces udenii*, *Mucor* sp., *Penicillium* sp. were the core fungi identified in dry-aged beef from five U.S. producers [8], while *Candida zeylanoides, Yarrowia alimentaria*, *Itersonilia pannonica*, *Mucor* complex *flavus*, and *Helicostylum elegans/pulchrum* were the main fungi in dry-aged beef from France [5].

**Figure 3:**
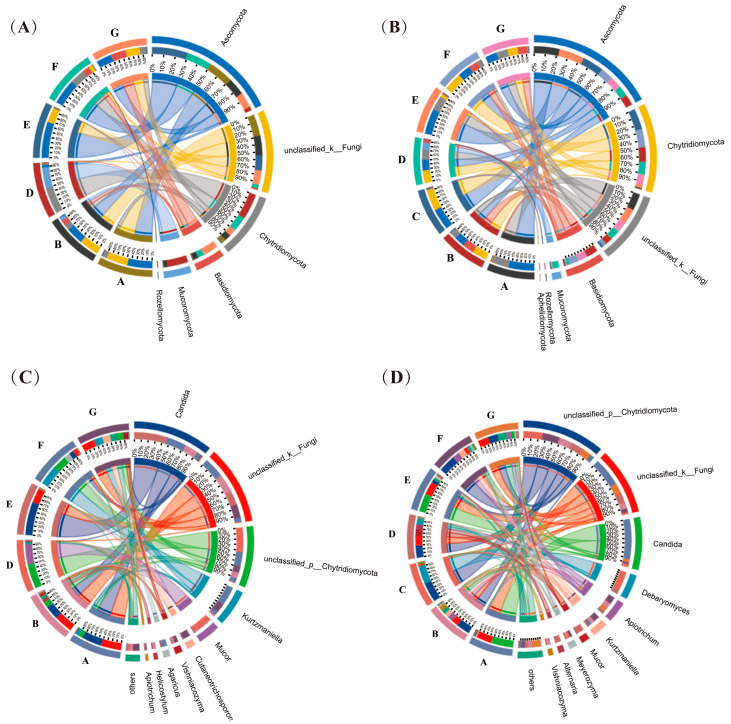
Circos plots of fungal community in dry-aged beef. (**A**) Fungal phyla in the crust, (**B**) fungal phyla in the interior, (**C**) fungal genera in the crust, (**D**) fungal genera in the interior. The letters (A, B, C, D, E, F, and G) in each circos plot represent different producers. Each plot displays the top 10 taxa.

The fungal composition of dry-aged beef is shown in Figure 3. The genus *Candida* was detected in all samples, with *C. sake* (62066 ASVs) and *C. alimentaria* (5971 ASVs) as the main *Candida* species (70373 ASVs). High proportions of *C. sake* were observed in samples from producers A (32.7% in the crust; 47.0% in the interior), B (21.5% in the crust), E (68.4% in the crust; 43.8% in the interior), and G (7.1% in the interior), while its presence in other samples was less than 5%. *C. alimentaria* was consistently detected at low levels (<10%) in all samples. *C. sake* was also identified by Coton et al. [5] as a key *Candida* species in dry-aged beef, along with other species such as *Candida zeylanoides* and *Candida glaebosa/pseudoglaebosa*.

*Kurtzmaniella* was identified in significant quantities in several samples, including the interior of samples from producer B (representing 10% of the genera), the interior of samples from producer E (representing 40%), the crust and interior of samples from producer F (representing 40% and 15%), and the crust of samples from producer G (representing 18%). *Kurtzmaniella* has been rarely reported in dry-aged beef. Phylum Chytridiomycota represented more than 5% in most samples, except for the crust of samples from producer E and the interior of samples from producer A. ASVs assigned to Chytridiomycota were only classified at the phylum level.

Several fungi species previously identified as core and dominant fungi in dry-aged beef were also detected in this study. However, they displayed relatively high proportions only in specific samples. For instance, *Debaryomyces* accounted for 45.1% in the interior of samples from producer C. *Mucor flavus* constituted 41% in the crust and 16% in the interior of samples from producer D. Low-abundance fungal genera, such as *Helicostylum*, *Apiotrichum*, *Alternaria*, and *Ascomycota*, were also present.

#### 3.3.3. Correlations Between Microbiota and Environmental Parameters

**Figure 4:**
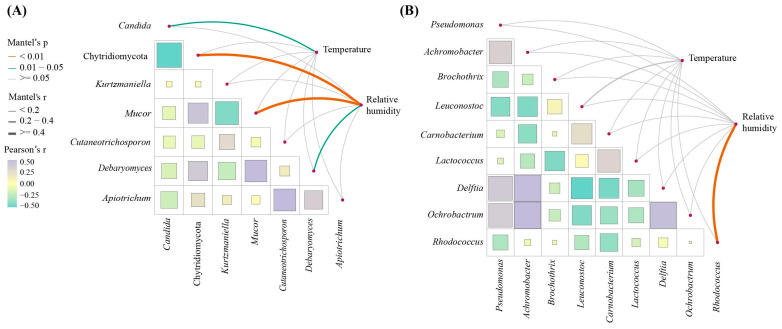
(**A**) The correlation between main fungal genera and environmental parameters (temperature and relative humidity); and (**B**) the correlation between main bacteria genera and environmental parameters (temperature and relative humidity). The color and width of the lines represent Mantel’s *p* value and Mantel’s r value. The yellow, green, and gray line represents *p* < 0.01, 0.01 < *p* < 0.05, and *p* ≥ 0.05, respectively.

Temperature and relative humidity (RH) are key environmental parameters that impact microbial growth. The correlations between main microbial genera and environmental parameters (temperature and RH) were analyzed using the Mantel test (Figure 4A,B). Within the temperature range of 0~2.5 °C, a significant correlation was observed between temperature and *Candida* (r = 0.29, *p* = 0.02). Within the RH range of 50%~85%, RH showed a significant correlation with *Rhodococcus* (r = 0.55, *p* = 0.007), *Mucor* (r = 0.85, *p* = 0.001), *Derbaryomyces* (r = 0.37, *p* = 0.03), and phylum Chytridiomycota (r = 0.65, *p* = 0.003). These findings demonstrated the importance of controlling temperature and relative humidity during the dry-aging process, as they may affect microbial communities, which subsequently affect the quality of dry-aged beef.

### 3.4. Enzymatic Potential of Microbiota in Dry-Aged Beef

**Figure 5:**
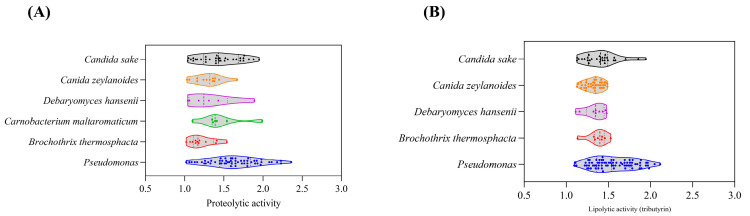
Proteolytic and lipolytic activities of fungi and bacteria isolates: (**A**) Proteolytic activities of isolates, and (**B**) lipolytic activities (tributyrin as the substrate) of isolates.

The cultural-dependent method detected only five bacterial genera and three fungal genera with proteolytic and lipolytic activities, despite high-throughput sequencing revealing high microbial diversity. Proteolytic and lipolytic activities varied significantly between and within genera and species (Figure 5A,B). Detailed results of microbial identification and enzymatic activities are provided in the Supplementary Materials (Tables S1 and S2).

Among the 129 bacterial isolates tested, 114 isolates showed enzymatic activities, containing *Pseudomonas* sp. (88 isolates), *B. thermosphacta* (13 isolates), *C. maltaromaticum* (7 isolates), *C. divergens* (2 isolates), *Moraxella osloensis* (3 isolates), and *Lactococcus carnosus* (1 isolates). *Pseudomonas* and *B. thermosphacta* were the most active bacteria and constituted the largest groups. *Pseudomonas* was identified in samples from all seven producers. As shown in Table 4, among the 88 *Pseudomonas* isolates tested, 51 displayed both proteolytic and lipolytic activities, and 40 exhibited the capacity to degrade both tributyrin and olive oil. Thirteen *B. thermosphacta* isolates were active, among which 12 isolates induced proteolysis, 8 isolates induced tributyrin lipolysis, 2 isolates induced olive oil lipolysis, and 7 isolates induced both proteolysis and tributyrin lipolysis. Proteolysis and lipolysis are regarded as the primary activities of *Pseudomonas* and *B. thermosphacta*, leading to their spoilage potentials [25,26].

Among the 113 fungal isolates tested, 93 isolates showed one or both tested activities, including 10 *Debaryomyces hansenii* isolates, 46 *Candida zeylanoides* isolates, 35 *Candida sake* isolates, and 2 *Yarrowia alimentaria* isolates (Table 4). None of the fungal isolates could degrade olive oil, although they were able to hydrolyze tributyrin. The result indicated that their lipases prefer the triacylglycerols of short-chain fatty acids to those of long-chain fatty acids.

*D. hansenii* and *C. zeylanoides* were commonly detected in dry-aged beef. They were also frequently and abundantly isolated from fermented sausages [27,28] and dry-cured meat products [29,30]. *D. hansenii* demonstrated positive effects on these products, including the production of free amino acids, free fatty acids, and volatile compounds [31,32,33], which aligned with its contributions to dry-aged beef [9,10].

*C. zeylanoides* was abundantly isolated from samples from producers A, B, G, and F, despite not being identified through the high-throughput sequencing approach. As *C. zeylanoides* belongs to the *Kurtzmaniella* (teleomorph) clade of the family Debaryomycetaceae [34], it may correspond to *Kurtzmaniella* detected by the high-throughput sequencing approach. *C. zeylanoides* is primarily characterized by its lipolytic activity [35]. Among the *C. zeylanoides* isolates, 73.9% featured exclusively lipolytic activity, and 21.7% displayed both proteolytic and lipolytic activity. Purriños et al. [31] reported that *C. zeylanoides* produced significant quantities of hydrocarbons in dry-cured “Lacón”, primarily through its hydrolytic activity on triglycerides, which facilitated the subsequent autoxidation of the released fatty acids. However, the contribution of *C. zeylanoides* to flavor development was minimal, as the high odor thresholds of hydrocarbons limited their sensory impact [31].

*C. sake* comprised a significant proportion of the fungal isolates, consistent with the high-throughput sequencing results. A large proportion (77.1%) of *C. sake* isolates featured both proteolytic and lipolytic activities. *C. sake* has been identified as a main yeast species in protein-rich food, such as Italian fermented sausages [36] and various types of cheese [37,38,39]. However, its association with meat aging or fermentation remains largely unexplored.

### 3.5. Hydrolytic Activities of Candida zeylanoides and Candida sake on Beef Protein and Fat

The hydrolytic potential of *C. zeylanoides* and *C. sake* on beef proteins and fat was further assessed using two strains with both high proteolytic and lipolytic activities. These strains were introduced into separate systems containing beef sarcoplasmic protein, myofibrillar protein, and fat. Proteolysis was monitored by determining contents of TCA-soluble peptides and free amino acids (FAAs), while lipolysis was evaluated by measuring the content of total free fatty acids (FFAs). As shown in Table 5, *C. zeylanoides* and *C. sake* significantly increased levels of TCA-soluble peptides in both myofibrillar and sarcoplasmic protein systems, demonstrating their abilities to degrade these proteins. In the sarcoplasmic protein system, *C. zeylanoides* and *C. sake* also led to an accumulation of FAAs, suggesting the effective action of their secreted exopeptidases. Conversely, in the myofibrillar protein system, *C. zeylanoides* and *C. sake* significantly (*p* < 0.05) reduced the total FAA content, likely due to their utilization of FAAs. Amino acid metabolism in yeast is associated with the production of various aromatic compounds, including higher alcohols, esters, and sulfur-containing compounds [40]. In the beef fat system, *C. zeylanoides* and *C. sake* increased total free fatty acid (FFA) levels by 33-fold and 12-fold, respectively, confirming their lipolytic potential. However, neither *C. zeylanoides* nor *C. sake* hydrolyzed olive oil, which also primarily comprises long-chain fatty acid esters, as determined by the agar diffusion method. The lack of activity may be attributed to differences in the long-chain fatty acid profiles of beef fat and olive oil, potentially influencing substrate specificity. The proteolytic and lipolytic activities, as well as the flavor production potential of *C. zeylanoides* and *C. sake*, require further investigation in real beef systems. It is also important to note that some *Candida* species, such as *Candida glabrata*, *Candida parapsilosis*, are opportunistic human pathogens [41]. Thus, both the quality effects and potential risks associated with *C. zeylanoides* and *C. sake* in dry-aged beef should be evaluated. *C. sake* isolated from kimchi has been demonstrated to be safe through genomic analysis, cellular assays, and in vivo animal study [42].

## 4. Conclusions

This study revealed variations in the microbiota of dry-aged beef; however, there are certain genera and species that constitute the majority of the microbiota. These key species warrant particular attention, as a large proportion of them exhibited strong proteolytic and lipolytic activities. The correlation between these microorganisms and sensory attributes of dry-aged beef should be explored in the future, to understand their roles in shaping its quality. Additionally, the limitations of this study highlight the need for further investigation. Specifically, as this study was conducted on samples ready for sale, it lacked information on the characteristics and quality of the raw beef, which may also influence the final microbiota. Future studies should incorporate those factors to provide a more comprehensive understanding of the factors influencing the microbiota of dry-aged beef.

## Figures and Tables

**Table 1:** Temperature and relative humidity in each producer.

	Temperature (°C)	Relative Humidity	Dry-Aging Time (Day)
Producer A	0 ± 1	60–75%	42
Producer B	—	—	42
Producer C	—	—	42
Producer D	2.0 ± 1.0	50%~55%	42
Producer E	2.5 ± 0.5	80%~85%	42
Producer F	1.5 ± 0.5	70%~80%	42
Producer G	2.0 ± 0.7	77%~78%	42

— means the information is not available.

**Table 2:** pH values and total volatile base nitrogen (TVB-N) values of dry-aged beef from different producers.

	pH Value	TVB-N Value (mg/100g)
Producer A	5.71 ± 0.05^ab^	13.67 ± 0.70^d^
Producer B	5.78 ± 0.02^a^	16.99 ± 0.70^b^
Producer C	5.66 ± 0.08^bc^	15.96 ± 0.56^bc^
Producer D	5.58 ± 0.06^c^	13.53 ± 1.07^d^
Producer E	5.63 ± 0.04^bc^	19.41 ± 1.13^a^
Producer F	5.68 ± 0.03^b^	10.12 ± 0.35^e^
Producer G	5.63 ± 0.02^bc^	14.37 ± 0.43^cd^

Different lowercase letters indicate significant differences between produces within a column (*p* < 0.05).

**Table 3:** Bacterial and fungal numbers in the crust and interior of dry-aged beef collected from different producers.

	Crust (log_10_ cfu/g)		Interior (log_10_ cfu/g)	
	Bacterial Numbers	Fungal Numbers	Bacterial Numbers	Fungal Numbers
Producer A	7.7 ± 0.4^b^	7.3 ± 0.9^a^	3.4 ± 0.3^c^	3.3 ± 0.2^c^
Producer B	7.3 ± 0.3^b^	5.9 ± 0.1^b^	6.3 ± 0.1^a^	5.5 ± 0.3^a^
Producer C	—	—	7.1 ± 0.3^a^	4.7 ± 0.1^b^
Producer D	6.2 ± 0.2^c^	5.8 ± 0.3^b^	3.3 ± 0.1^c^	3.1 ± 0.2^c^
Producer E	8.8 ± 0.4^a^	7.7 ± 0.3^a^	4.7 ± 0.7^b^	3.0 ± 0.3^c^
Producer F	8.3 ± 0.2^a^	6.2 ± 0.4^b^	2.5 ± 0.4^c^	<2.0
Producer G	7.3 ± 0.3^b^	5.3 ± 0.2^b^	<2.0	<2.0

Different lowercase letters indicate significant differences between producers within a column (*p* < 0.05). Microbial numbers in the crust of dry-aged beef from producer C were not analyzed.

**Table 4:** Numbers of proteolytic and lipolytic isolates in different bacteria or fungi species.

Bacteria/Fungi Species	Total Number of Isolates	Proteolytic Isolates	Lipolytic (Tributyrin) Isolates	Lipolytic (Olive Oil) Isolates	Both Proteolytic and Lipolytic (Tributyrin) Isolates
*Pseudomonas* sp.	88	55	81	40	51
*Brochothrix thermosphacta*	13	12	8	2	7
*Carnobacterium maltaromaticum*	7	7	1	0	1
*Carnobacterium divergens*	2	1	1	1	0
*Moraxella osloensis*	3	1	3	2	1
*Lactococcus carnosus*	1	1	0	0	0
*Candida sake*	35	33	29	0	27
*Candida zeylanoides*	46	12	44	0	10
*Debaryomyces hansenii*	10	6	9	0	5
*Yarrowia alimentaria*	2	1	2	0	1

**Table 5:** TCA-soluble peptides, total free amino acids, and total free fatty acids of non-inoculated and inoculated groups in beef protein system and fat system.

	Myofibrillar Protein		Sarcoplasmic Protein		Fat
	TCA-Soluble Peptides (mg/mL)	Total Free Amino Acids (μmol/mL)	TCA-Soluble Peptides (mg/mL)	Total Free Amino Acids (μmol/mL)	Total Free Fatty Acid (μmol/g)
Non-inoculated	0.316 ± 0.009^c^	8.601 ± 0.155^a^	0.515 ± 0.005^c^	2.582 ±0.254^c^	0.690 ± 0.445^c^
*Candida zeylanoides*	0.953 ± 0.028^a^	2.560 ± 0.207^c^	0.794 ± 0.007^b^	4.114 ± 0.103^b^	23.867 ± 1.305^a^
*Candida sake*	0.536 ± 0.003^b^	4.703 ± 0.239^b^	0.987 ± 0.004^a^	6.823 ± 0.034^a^	9.223 ± 1.811^b^

Different lowercase letters indicate significant differences between groups within a column (*p* < 0.05).

## Data Availability

The original contributions presented in the study are included in the article/Supplementary Materials, further inquiries can be directed to the corresponding author.

## Supplementary Materials

The following supporting information can be downloaded at: https://www.mdpi.com/article/10.3390/foods14030529/s1, Table S1: Identification of proteolytic and lipolytic bacteria isolates from dry-aged beef; Table S2: Identification of proteolytic and lipolytic fungi isolates from dry-aged beef.
