# Supplementary material for: Insights into Microbial Community and Its Enzymatic Profiles in Commercial Dry-Aged Beef
Source: Foods. 2025 Feb 6;14(3):529. doi: 10.3390/foods14030529 (PMC11817974; doi:10.3390/foods14030529)
Supplement: Supplementary file 1 [file foods-14-00529-s001.zip › Table S1.pdf]

Table S1 Identification of proteolytic and lipolytic bacteria isolates from dry-aged beef

Putative identification	Accession number	Proteolysis	Lipolysis		Origins
			Tributyrin	Olive oil	
Pseudomonas sp.	-	++	+++	++	Producer A
		+	+	-	
		+	-	-	
		+	-	-	
		-	++	++	
		++	++	-	Producer B
		+	+++	++	
		+++	++	++	
		+	-	-	
		-	+	-	
		-	+	+	
		++	+	+	
		++	+	+	
		++	++	+	
		++	++	+	
		++	++	+	
		+	+	-	
		-	+	-	
		+	+	-	
		+	+	-	
		++	++	-	
		++	++	+	
		++	++	+	
		++	++	+	
		-	+	-	Producer C
		++	+	-	
		++	++	+	
		++	++	+	
		++	++	-	
		-	++	+	Producer D
		-	++	+	
		++	+	-	
		++	++	+	
		++	++	+	
		++	+	-	
		+++	++	+	
		+	-	-	Producer E
		+	++	+	
		+	++	+	
		-	++	+	

-	++	+	
-	++	++	
-	+	-	
-	+	-	
-	+	-	
-	+	-	
-	+	+	
++	+	+	
++	++	+	
++	++	+	
++	++	-	
++	++	-	
++	++	-	
++	++	-	
++	++	-	
++	++	+	Producer F
+	+	+	
-	+	-	
-	-	-	
-	+	-	
+	++	-	
+++	++	-	
+	+	-	
-	-	-	
-	+	-	
-	+	-	
-	+	-	
-	+	-	
-	+	-	
-	+	-	
-	+	-	
-	-	-	
-	+	-	
-	+	-	
-	++	++	Producer G
-	++	++	
++	+	+	
-	+	-	
++	++	+	
++	++	-	
++	+	-	
+++	+++	+	
+++	+	+	

		+	++	++	
		+	+	++	
		+	+	+	
		-	++	-	
		+++	++	-	
Brochothrix thermosphacta	AODI01000055	+	+	-	Producer A
		-	+	-	
		+	+	++	
		+	+	-	Producer C
		+	+	-	Producer E
		+	+	-	
		+	-	-	
		+	-	-	
		++	++	++	Producer G
		+	+	-	
		+	-	-	
		+	-	-	
		+	-	-	
Carnobacterium divergens	JQLO01000001	-	+	+	Producer A
		+	-	-	Producer E
Carnobacterium maltaromaticum	JQMX01000001	+	+	-	Producer F
		+	-	-	
		++	-	-	
		+	-	-	
		++	-	-	
		+	-	-	
		+	-	-	
Moraxella osloensis	CP014234	+++	+	+	Producer D
		-	+	+	
		-	+	-	Producer G
Lactococcus carnosus	MT772277	+	-	-	Producer F

Enzymatic activity was represented by the ratio of the diameter of the halo to the diameter of the colony. -, no activity; +, weak activity (1 < ratio ≤1.5); ++, moderate activity (1.5 < ratio ≤2); strong activity (2 < ratio).
