# Supplementary material for: Insights into Microbial Community and Its Enzymatic Profiles in Commercial Dry-Aged Beef
Source: Foods. 2025 Feb 6;14(3):529. doi: 10.3390/foods14030529 (PMC11817974; doi:10.3390/foods14030529)
Supplement: Supplementary file 1 [file foods-14-00529-s001.zip › Table S2.pdf]

Table S2 Identification of proteolytic and lipolytic fungi isolates from dry-aged beef

Putative identification	Accession number	Proteolysis	Lipolysis		Origins
			Tributyrin	Olive oil	
Debaryomyces hansenii	MH545920.1	-	+	-	Producer C
		+	-	-	Producer D
		++	+	-	
		+	+	-	
		-	+	-	
		-	+	-	
		-	+	-	
		+	+	-	
		+	+	-	
		+	+	-	
Candida zeylanoides	EF687773.1	+	+	-	Producer A
	OW987024.1	+	+	-	Producer A
	OP216879.1	+	-	-	Producer A
	KX376266.1	-	+	-	Producer B
		-	+	-	Producer G
	KX394416.1	-	+	-	Producer B
		-	+	-	Producer B
		+	+	-	Producer B
		-	+	-	Producer F
		-	+	-	Producer F
		-	+	-	Producer F
		-	+	-	Producer F
		-	+	-	Producer G
	KY106918.1	+	+	-	Producer B
		-	+	-	Producer F
		-	+	-	Producer F
		-	+	-	Producer F
		-	+	-	Producer G
		+	+	-	Producer G
	MH459420.1	++	-	-	Producer B
		-	+	-	Producer B
		-	+	-	Producer F
		-	+	-	Producer F
		-	+	-	Producer F
		-	+	-	Producer F
		-	+	-	Producer F
		-	+	-	Producer F
		+	+	-	Producer F
		+	+	-	Producer F

		+	+	-	Producer F
		-	+	-	Producer G
		-	+	-	Producer G
		-	+	-	Producer G
		-	+	-	Producer G
		+	+	-	Producer G
	MT163366.1	-	+	-	Producer F
	MK613256.1	-	+	-	Producer F
		-	+	-	Producer G
	HE660058.1	-	+	-	Producer F
	KX385848.1	-	+	-	Producer F
		+	+	-	Producer F
		-	+	-	Producer G
	KY102539.1	-	+	-	Producer G
		-	+	-	Producer G
		-	+	-	Producer G
	LT594199.1	-	+	-	Producer G
Candida Sake	MT269550.1	++	++	-	Producer A
		+	+	-	Producer A
		-	+	-	Producer E
		+	+	-	Producer E
		+	+	-	Producer E
		+	-	-	Producer E
	MK394102.1	++	+	-	Producer A
		++	+	-	Producer A
		++	+	-	Producer A
		+	+	-	Producer A
		++	+	-	Producer B
		++	+	-	Producer B
		++	+	-	Producer B
		++	+	-	Producer B
		+	++	-	Producer E
		+	++	-	Producer E
		+	++	-	Producer E
		+	++	-	Producer E
		-	++	-	Producer E
		+	+	-	Producer E
		+	+	-	Producer E
		+	+	-	Producer E
		+	+	-	Producer E
		+	+	-	Producer E
		+	+	-	Producer E
		+	+	-	Producer E

		+	+	-	Producer E
		+	-	-	Producer E
		+	-	-	Producer E
		+	-	-	Producer E
		+	-	-	Producer E
		+	-	-	Producer E
		++	+	-	Producer F
		+	+	-	
		+	+	-	Producer G
Yarrowia alimentaria	MH595235.1	+++	+	-	Producer A
		-	+	-	

Enzymatic activity was represented by the ratio of the diameter of the halo to the diameter of the colony. -, no activity; +, weak activity (1 < ratio ≤1.5); ++, moderate activity (1.5 < ratio ≤2); strong activity (2 < ratio).
